# Biochemical Screening of the New Strain *Dolichospermum solitarium* (Cyanobacteria) for Phytoremediation and Biotechnology

**DOI:** 10.3390/ijms27104336

**Published:** 2026-05-13

**Authors:** Irina Maltseva, Aleksandr Yakoviichuk, Svetlana Maltseva, Maxim Kulikovskiy, Yevhen Maltsev

**Affiliations:** 1 Faculty of Natural Sciences, Melitopol State University, Melitopol 272312, Russia; 2 K.A. Timiryazev Institute of Plant Physiology RAS, IPP RAS, Moscow 127276, Russia

**Keywords:** antioxidant enzymes, Cyanobacteria, carotenoids, chlorophyll, productivity, retinol, α-tocopherol

## Abstract

This work described a novel freshwater strain of Cyanobacteria, *Dolichospermum solitarium* MAC–C17 from the Zaporozhye region in the south of European Russia and characterised its growth and biochemical parameters. The strain demonstrated high biomass, lipid, and protein productivity, comparable to the best-known data for high-productivity cyanobacteria. The highest protein content (383.76 mg g^−1^ dry weight) was in the exponential growth phase. In contrast, the highest content of lipids, vitamin E (α-tocopherol) and vitamin A (retinol) was in the stationary phase (150.05 mg g^−1^, 45.58 µg g^−1^ and 31.61 µg g^−1^). The productivity of *Dolichospermum solitarium* MAC–C17 was 19.26 mg L^−1^ d^−1^ for lipids, 42.54 mg L^−1^ d^−1^ for protein, 4.06 µg L^−1^ d^−1^ for retinol, and 5.85 µg L^−1^ d^−1^ for α-tocopherol. A distinctive feature of the new strain was its high chlorophyll *a* content (4.87–6.12 mg g^−1^). The antioxidant defence system of *Dolichospermum solitarium* differed between the exponential and stationary growth phases. During the exponential growth phase, strain MAC–C17 exhibited high catalase activity. In contrast, the stationary phase was characterised by a distinct shift in its antioxidant profile, marked by a substantial increase in superoxide dismutase and glutathione peroxidase activities, a significant rise in retinol and α-tocopherol levels. These findings suggest that strain *Dolichospermum solitarium* MAC–C17 possessed considerable biotechnological potential. It demonstrated strong potential as a producer of high-value commercial compounds, a promising candidate for developing antioxidant formulations, and a valuable asset for applications that boost agricultural yields and advance phytoremediation technologies.

## 1. Introduction

Cyanobacteria are the most ancient group of photosynthetic organisms, playing a crucial role in oxygenating the atmosphere and shaping early biogeochemical cycles [1]. Among Cyanobacteria, some species combine the ability to photosynthesise with the capacity to fix atmospheric nitrogen. Thus, they play a vital role not only in the Earth’s carbon cycle but also in its nitrogen cycle [2]. Cyanobacteria have a long history of adaptation to diverse environments. Currently, they live in a wide variety of habitats: freshwater and saltwater reservoirs, hot springs, on the surface and in deep soil horizons, on tree bark, rock, and ice surfaces [3,4]. Cyanobacteria can survive at high and low temperatures, in direct sunlight, and in deep soil layers, endure periodic desiccation, and withstand high doses of radiation [5,6,7,8]. Cyanobacteria are producers of organic matter and play a significant role in sustaining food webs in modern ecosystems. Nitrogen-fixing species serve as a source of biologically available nitrogen for the growth of other organisms in nitrogen-poor ecosystems [9,10].

The unique ecological, physiological, and biochemical characteristics of Cyanobacteria have generated significant interest in studying and utilising these organisms in practice. Earlier, Maltsev et al. [8] noted that cyanobacteria improve soil structure and fertility and initiate primary soil formation processes in various industrial landfills. They survive on substrates containing many heavy metals and other toxic compounds, and many researchers have noted their use in phytoremediation technologies [8,9,11]. There is known positive experience in using Cyanobacteria to restore soil fertility and enhance agricultural productivity [9,12], and in technologies for wastewater treatment from various pollutants [13]. As a source of lipids, proteins, polyunsaturated fatty acids, carotenoids (Car), vitamins, enzymes, and other valuable compounds, Cyanobacteria are in demand in pharmacology, the food industry, and biodiesel production [9,14,15,16,17]. It is important to note that in eutrophic water bodies, Cyanobacteria can proliferate massively, causing so-called ‘algal blooms’, which have negative ecological consequences, degrade water quality, and pose risks to the functioning of other aquatic organisms [18,19].

Thus, research into the strategies underlying cyanobacteria’s adaptive capabilities is necessary. This would allow us to control their growth, either by limiting it in eutrophic water bodies or by stimulating it for phytoremediation and the production of commercially valuable compounds in biotechnology. It is well known that strong exposure to one or more factors disrupts the redox balance of cells by generating significant amounts of reactive oxygen species (ROS). The antioxidant system protects cells and counteracts the oxidative damage caused by ROS. The antioxidant system functions through low-molecular-weight and high-molecular-weight antioxidants [20]. Low-molecular-weight compounds, such as α-tocopherol, vitamin A, glutathione, etc., reduce the rate of free radical generation and decrease the concentration of oxidative reaction products. High-molecular-weight antioxidants include various enzymes, such as superoxide dismutase (SOD), catalase (CAT), and glutathione peroxidase (GPx) [21,22]. The restoration of cellular redox balance occurs through a coordinated system of interactions between low- and high-molecular-weight antioxidants [23]. Stressful exposure that exceeds the capacity of the cellular antioxidant system can lead to cell death. Therefore, one of the most important problems to be solved is establishing the features of the antioxidant defence system in Cyanobacteria that enable them to maintain viability under various external influences.

Heterocytous species with single trichomes, grouped in the genus *Anabaena* Bory ex Bornet et Flahault, often live in various aquatic and terrestrial habitats. These species, along with other cyanobacteria, can cause water blooms; some live in soils, hot waters, and saline biotopes and are highly resistant to abiotic stresses [24,25]. From a practical use perspective, *Anabaena*, as a nitrogen-fixing organism, holds great potential for developing technologies to enrich agricultural fields with nitrogen compounds [12]. *Anabaena* species are known for their antibacterial activity [26] and ability to synthesise lipids, carotenoids, and other biotechnologically valuable compounds [14,15,16,17]. Researchers have paid considerable attention to studies of growth dynamics, photosynthesis, and the accumulation of biotechnologically valuable compounds in *Anabaena* biomass, as well as the search for strategies to increase its productivity [24]. However, at present, there is limited information on the features of the antioxidant protection system in cyanobacteria [27,28].

A recent analysis of *Anabaena* diversity, considering phenotypic and molecular features, confirmed the heterogeneity of this genus [29]. Planktonic species isolated into a separate genus, *Dolichospermum* (Bornet et Flahault) P.Wacklin, L.Hoffmann et Komárek [29,30]. While exploring the cyanobacterial flora of the Molochnaya River, we isolated a novel cyanobacterial strain with *Dolichospermum*-like morphology. The ability of most *Dolichospermum* species to accumulate lipids, proteins, and vitamins has not yet been studied, and research combining assessments of metabolite synthesis and antioxidant system activity is virtually absent for Cyanobacteria in general.

This study focused on the combined analysis of morphological (light microscopy) and molecular (phylogeny of the 16S rRNA gene) data, and on the screening of lipid, protein, α-tocopherol, and vitamin A content, as well as the antioxidant status, of the new strain *Dolichospermum solitarium* (Klebahn) Wacklin, L.Hoffmann et Komárek.

## 2. Results

### 2.1. Strain Description

#### *Dolichospermum solitarium* MAC–C17

**Figure 1:**
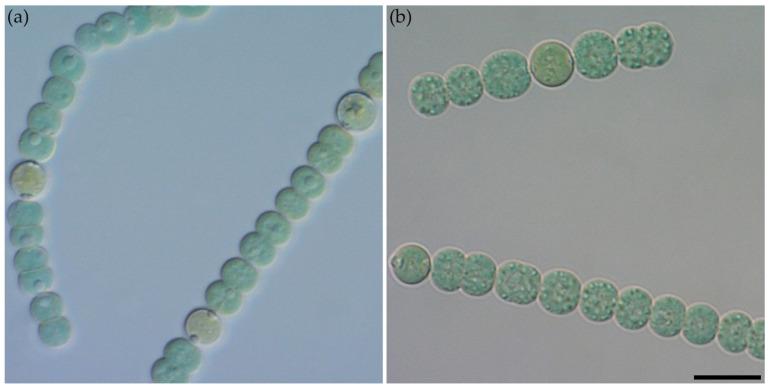
Morphological characteristics of *Dolichospermum solitarium* MAC–C17. Nomarski interference micrographs, scale bar = 10 μm. Individual filaments with intercalary heterocytes, age 1 week (**a**). Mature filaments with gas vesicles, age 3 weeks (**b**).

Morphological description (Figure 1): Trichomes are isopolar, with constrictions at cross-walls, without firm sheaths. Apical cells are morphologically similar to vegetative cells. Vegetative cells are spherical or slightly oval-elongated, blue-green to brown-green, 6.0–8.2 μm in diameter, with gas vesicles. The cell wall is thin. Intercalary heterocytes are brown-green to brownish, solitary or in pairs, spherical, and 6.0–7.5 in diameter. Akinetes are intercalary, large, long-cylindrical, 22.0–29.0 μm long and 11.0–14.0 μm wide, and solitary or successively in rows. The filaments are solitary or in small clusters.

Habitat: The strain MAC–C17 was isolated from a planktonic sample collected in the Molochnaya River located in the Zaporozhye region (N 46°57′05″, E 35°285′20″). The water temperature was 18 °C with a pH of 7.85.

Sequence data: GenBank accession PZ385169 for the 16S rRNA gene partial sequence.

**Figure 2:**
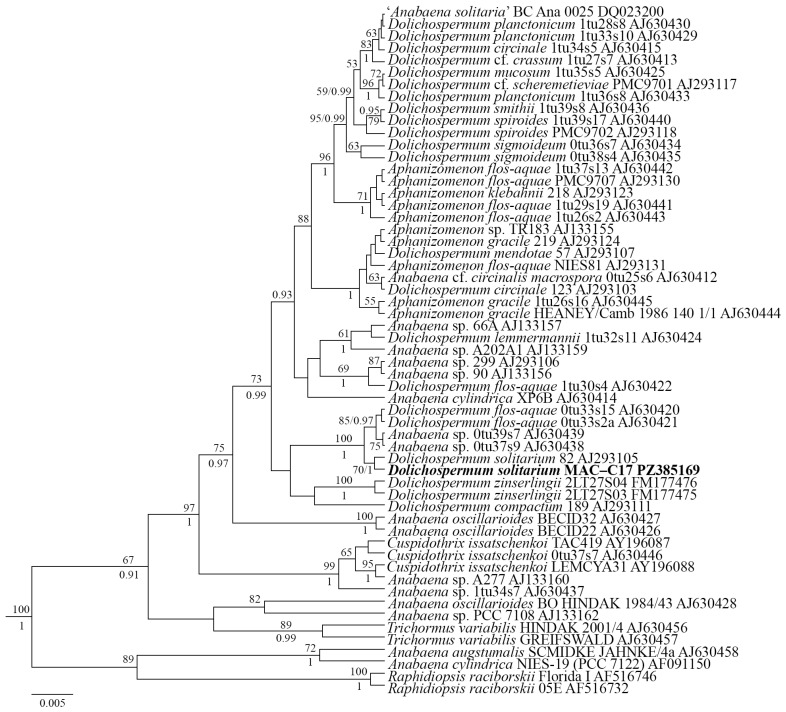
Phylogenetic position of the new *Dolichospermum solitarium* strain (indicated in bold) within *Anabaena*, *Dolichospermum*, and *Aphanizomenon* Morren ex Bornet et Flahault genera, based on Bayesian inference for the partial 16S rRNA gene. The total length of the alignment is 869 characters. Values above the horizontal lines are bootstrap support (>50%) from ML analyses; values below the horizontal lines (or to the right of the slash) are Bayesian posterior probabilities (>0.9). Strain numbers (if available) and GenBank accession numbers are indicated for all sequences.

Molecular analysis: The 16S rDNA phylogenetic tree was constructed using 56 nucleotide sequences of closely related cyanobacterial strains from the National Centre for Biotechnology Information (NCBI) and the novel strain’s sequence. Reconstruction of phylogenetic relationships using maximum likelihood (ML, where BS is the bootstrap support) and Bayesian (BI, where PP is the posterior probability) methods showed that all strains classified at the genus level had a clear separation into different clades (Figure 2). MAC–C17 formed a common clade with the strain *D. solitarium* 82 with BS 70 and PP 1.0. Comparative analysis of partial 16S sequences (869 bp) for the newly identified MAC–C17 vs. the strain *D. solitarium* 82 revealed several evolutionary events. MAC–C17 sequence differed by two transitions (A→G and G→A) and ‘G’, ‘C’ and ‘T’ deletions.

Strain MAC–C17 was classified as belonging to the species *Dolichospermum solitarium*, genus *Dolichospermum*, family Aphanizomenonaceae, order Nostocales and class Cyanophyceae based on morphology and molecular data.

### 2.2. Growth and Biochemical Characteristics

In this study, the strain *D. solitarium* MAC–C17 demonstrated a specific growth rate of 0.12 d^−1^ and a volumetric biomass productivity of 128.43 mg L^−1^ d^−1^. The strain reached the stationary phase on the 15th day of cultivation.

**Figure 3:**
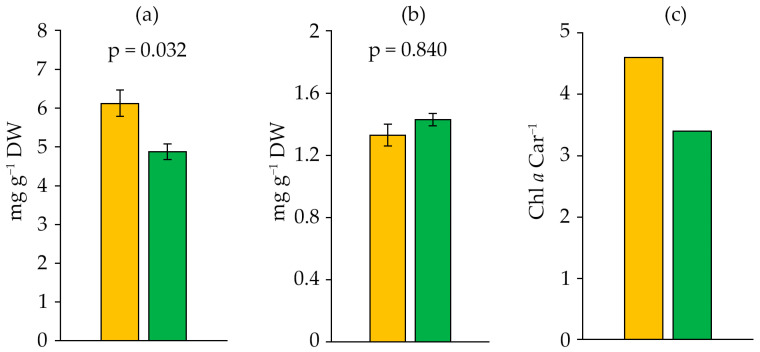
Chl *a* (**a**), carotenoids (**b**) and Chl *a* Car^−1^ ratio (**c**) in *Dolichospermum solitarium* MAC–C17 biomass. Colours correspond to the data for orange—logarithmic growth phase; green—stationary growth phase. The difference between logarithmic and stationary growth phases was significant at *p* ≤ 0.05 (M ± SD, *n* = 3).

The chlorophyll *a* (Chl *a*) and carotenoid content differed between the logarithmic and stationary growth phases (Table 1). The stationary phase was characterised by a 20.4% reduction of Chl *a* content (Figure 3a). The carotenoid content did not significantly increase in the stationary phase compared to the logarithmic phase (Figure 3b). The Chl *a* Car^−1^ ratio shifted in the stationary phase towards an increased proportion of carotenoids and a reduced role of Chl *a* (Figure 3c).

**Figure 4:**
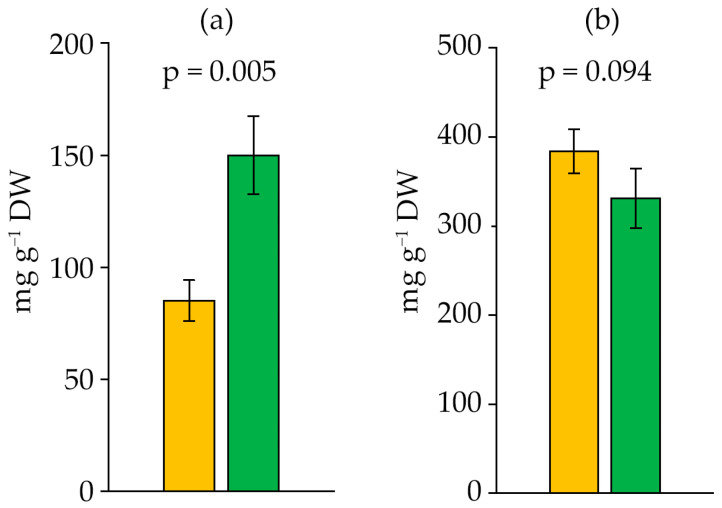
Lipids (**a**) and protein (**b**) content in *Dolichospermum solitarium* MAC–C17 biomass. Colours correspond to the data for orange—logarithmic growth phase; green—stationary growth phase. The difference between logarithmic and stationary growth phases was significant at *p* ≤ 0.05 (M ± SD, *n* = 3).

The lipid content in the biomass of *D. solitarium* MAC–C17 increased sharply in the stationary phase, reaching 150.05 mg g^−1^ dry weight (DW), which was almost twice as high as in the logarithmic phase (Figure 4a). The protein content did not change significantly during the strain’s growth (Figure 4b).

**Figure 5:**
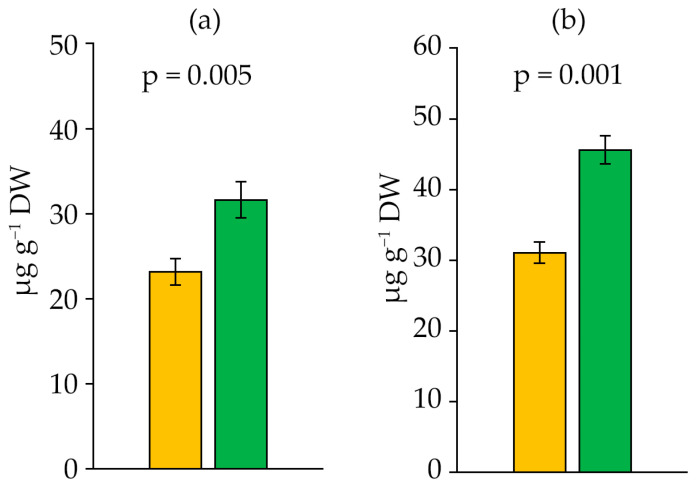
Retinol (**a**) and α-tocopherol (**b**) content in *Dolichospermum solitarium* MAC–C17 biomass. Colours correspond to the data for orange—logarithmic growth phase; green—stationary growth phase. The difference between logarithmic and stationary growth phases was significant at *p* ≤ 0.05 (M ± SD, *n* = 3).

In the stationary phase, there was an increase in the content of retinol and α-tocopherol relative to the logarithmic phase by 36.5% and 46.7% (Figure 5), respectively.

The productivity of *D. solitarium* MAC–C17 was 19.26 mg L^−1^ d^−1^ for lipids, 42.54 mg L^−1^ d^−1^ for protein, 4.06 µg L^−1^ d^−1^ for retinol, and 5.85 µg L^−1^ d^−1^ for α-tocopherol.

### 2.3. SDH and Antioxidant Enzyme Activity

**Figure 6:**
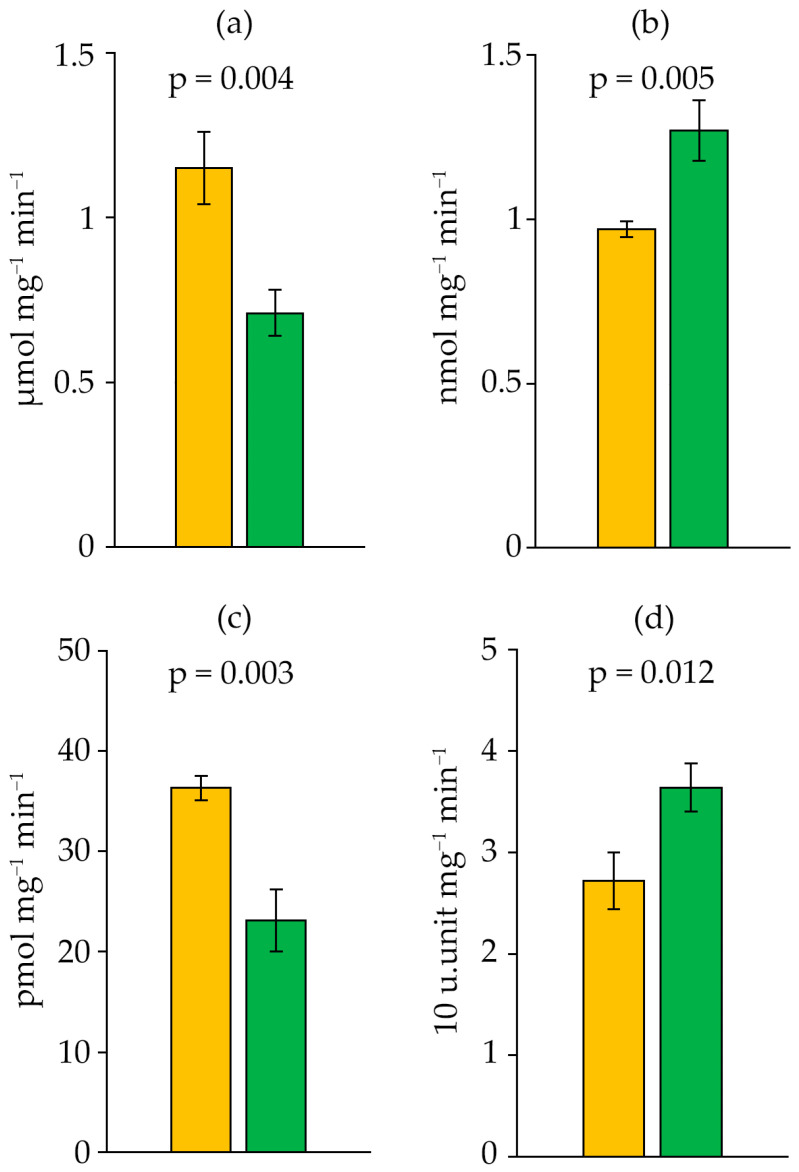
SDH (**a**), GPx (**b**), CAT (**c**) and SOD (**d**) activity in *Dolichospermum solitarium* MAC–C17 biomass. Colours correspond to the data for orange—logarithmic growth phase; green—stationary growth phase. The difference between logarithmic and stationary growth phases was significant at *p* ≤ 0.05 (M ± SD, *n* = 3).

Succinate dehydrogenase (SDH) activity decreased by 1.6-fold with the age of the *D. solitarium* MAC–C17 culture (Figure 6a). This indicated more intensive energy metabolism in the strain’s cells during the logarithmic growth phase than in the stationary phase.

During the growth of *D. solitarium* MAC–C17, an increase in GPx and SOD activity was observed in the stationary phase compared to the logarithmic phase, by 30.9% and 33.8%, respectively (Figure 6b,c). Conversely, CAT activity decreased by 36.4% in the stationary phase (Figure 6d).

### 2.4. Intensity of Lipid Peroxidation Processes at Different Growth Phases

**Figure 7:**
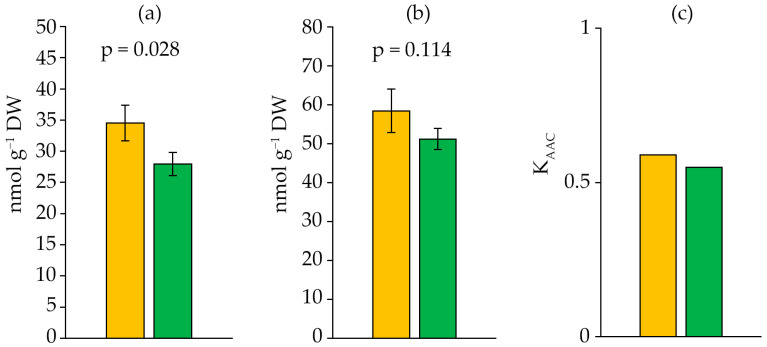
TBARS (**a**), TBARS_in_ (**b**) content and K_AAC_ (**c**) in *Dolichospermum solitarium* MAC–C17 biomass. Colours correspond to the data for orange—logarithmic growth phase; green—stationary growth phase. The difference between logarithmic and stationary growth phases was significant at *p* ≤ 0.05 (M ± SD, *n* = 3).

The concentration of thiobarbituric-acid-reactive substance (TBARS) decreased with the age of the culture by 1.24-fold before the initiation of lipid peroxidation (LPO) and by 1.14-fold after the initiation of LPO by Fe^2+^ ions (TBARS_in_) (Figure 7a,b). The strain had an antioxidant activity coefficient (K_AAC_) with a lower value in the stationary growth phase (Figure 7c).

## 3. Discussion

Wacklin et al. [29] proposed the genus *Dolichospermum* as a new nomenclatural unit for the planktonic species *Anabaena*, whose morphotype is characterised by gas vesicles. At the same time, the authors themselves noted that the genus *Dolichospermum* is not monophyletic. New research has confirmed that *Anabaena*, *Dolichospermum*, and *Aphanizomenon* are grouped in phylogenetic analysis to form the ‘clade *Anabaena*, *Dolichospermum*, and *Aphanizomenon* (ADA)’ [31]. Molecular and phenotypic variability within species of this clade remains a topic of ongoing discussion. The taxonomic categorisation of these genera, as well as the alignment of morphological criteria with phylogenetic analysis results, remains ambiguous [19,30,32,33,34].

Analysis of the morphological features of the strain MAC–C17 showed a high similarity to those of the genus *Dolichospermum*. The common features were filaments straight, solitary, or sometimes in clusters; trichomes with almost spherical cells, intercalary spherical heterocytes, and cylindrical akinetes [35]. At the same time, strain MAC–C17 was characterised by smaller sizes of heterocytes (up to 7.5 μm in diameter compared to 10.0 μm in diagnosis) and length of akinetes (22.0–29.0 μm compared to 28.0–35.0 μm) [36].

Phylogenetic analysis using the ML and BI methods with the 16S rRNA gene showed that strain MAC–C17 was robustly placed within the ADA group and, first of all, to the strain *D*. *solitarium* 82 collected in Lake Karpjärvi, Finland [37]. A sister position to *D. solitarium* strains was shown by the strains 0tu33s15 and 0tu33s2a of *Dolichospermum flos-aquae* (Bornet et Flahault) P.Wacklin, L.Hoffmann et Komárek with strains *Anabaena* sp. 0tu37s9 and 0tu39s7 collected in Lake Tuusulanjärvi, Finland [38]. The differences between the MAC–C17 and these strains were a length of akinetes up to 29.0 μm, versus 13.5–22.0 μm in *D*. *flos-aquae* 0tu33s15 and 11.6–18.6 in *D*. *flos-aquae* 0tu33s2a; large width of trichomes (up to 8.4 and 9.0 μm) in *Anabaena* sp. 0tu37s9 and 0tu39s7, respectively. *Dolichospermum zinserlingii* (Kossinskaja) Wacklin, Hoffmann et Komárek and *Dolichospermum compactum* (Nygaard) P.Wacklin, L.Hoffmann et J.Komárek strains had the closest phylogenetic position to *D. solitarium*, albeit with low bootstrap support in MP and BI. Morphologically, *D. zinserlingii* differed from adjacent akinetes to heterocytes [35]. Smaller sizes of vegetative cells, heterocytes and akinetes were distinct from *D. compactum* from MAC–C17. Generally, the tree topology corresponded well with previously obtained phylograms [29,38]. One of the exceptions was the position of the strain ‘*Anabaena solitaria*’ BC Ana 0025, which had a sister position to *Dolichospermum planctonicum* (Brunnthaler) Wacklin, L.Hoffmann et Komárek strains (Figure 2). Such genetic data imply the possible discrepancy between the strain BC Ana 0025 and *D*. *solitarium*.

*D. solitarium* (formerly *Anabaena solitaria* Klebahn) is a widely distributed nitrogen-fixing cyanobacterial species in various freshwater reservoirs and is frequently reported among species causing water ‘blooms’ [30,39,40].

The pigment system of Cyanobacteria, which provides photosynthesis, contains only one form of chlorophyll, chlorophyll *a*, along with pigments such as carotenoids and phycobiliproteins [41]. The Chl *a* content in *D. solitarium* MAC–C17 was 4.87–6.12 mg g^−1^ DW. This is higher than in *Anabaena sphaerica* Bornet et Flahault MBDU 105 (approximately 1.65 mg g^−1^ DW) [42], *Nostoc* sp. (approximately 0.73 mg g^−1^ DW) [43], and in *Bracteacoccus minor* (Schmidle ex Chodat) Petrová MZ–Ch31 (approximately 2.75 mg g^−1^ DW of total chlorophylls) [44], but lower than in *Chlorococcum infusionum* (Schrank) Meneghini TISTR 8461, which contained 8.45 mg g^−1^ DW of total chlorophylls [45]. In terms of carotenoid content, *D*. *solitarium* MAC–C17 surpassed *Synechocystis* sp. and *Anabaena sphaerica* MBDU 105, characterised by 4.5–3.8 µg g^−1^ DW and 0.5 µg g^−1^ DW of carotenoids, respectively [42,46], but was lower than in some green microalgae, such as *Chlorococcum infusionum* TISTR 8461 and *Bracteacoccus aggregatus* Tereg BM5/15, which contained 4.14 mg g^−1^ DW and 6.3 mg g^−1^ DW of carotenoids, respectively [45,47]. Such variability in pigment content is associated with both specific strain characteristics and cultivation parameters [41].

The higher Chl *a* content in the exponential growth phase of *D. solitarium* MAC–C17 indicates increased metabolic and photosynthetic activity during this phase compared to the stationary phase. Active photosynthesis is associated with the generation of significant amounts of ROS [48] and enhanced LPO. In the exponential growth phase, SDH activity was higher. SDH is not a direct component of the antioxidant system (AOS), but its activity affects the AOS, as ROS are generated at specific SDH sites [49]. SDH is associated with ATP synthesis through the respiratory chain. In the tricarboxylic acid cycle, SDH also catalyses the oxidation of succinate to fumarate, reducing ubiquinone (coenzyme Q10) to ubiquinol. The antioxidant effect of ubiquinol is related to its ability to neutralise free radicals [50]. Thus, SDH activity plays a vital role in maintaining the cellular redox balance.

In the exponential growth phase, the amount of TBARS and TBARS_in_ in *D. solitarium* MAC–C17 was higher than in the stationary phase. In addition, during the exponential phase, CAT activity was higher, indicating an active conversion of H_2_O_2_ into H_2_O and O_2_. At the same time, SOD and GPx activities were higher during the stationary growth phase. This likely reflects the specific features of metabolic adaptations and ROS protection mechanisms in this strain. As the culture grows, nutrient depletion creates stressful growth conditions, leading to increased activity of the antioxidant enzymes SOD and GPx. Earlier studies on cyanobacteria and microalgae grown under various abiotic stresses, including nitrogen starvation, have also reported increased activity of antioxidant enzymes [51,52,53]. Modulation of non-enzymatic antioxidants was also observed in the stationary growth phase—the content of retinol and α-tocopherol increased. Low-molecular-weight antioxidants, including vitamins, can help limit the development of lipid peroxidation reactions associated with oxidative stress. Both α-tocopherol (the most bioactive form of vitamin E) and retinol (the most bioactive form of vitamin A) possess high antioxidant potential [54]. The α-tocopherol content in cyanobacteria is generally low. According to Mudimu et al. [55], the maximum amounts of α-tocopherol in the stationary phase were found in *Limnospira maxima* (Setchell et N.L.Gardner) Nowicka-Krawczyk, Mühlsteinová et Hauer and *Limnospira platensis* (Gomont) K.R.S.Santos et Hentschke—177.23 and 146.48 µg g^−1^ DW, respectively—and in *Aphanizomenon gracile* Lemmermann and *Aphanizomenon flos-aquae* Ralfs ex Bornet et Flahault—143.17 and 101.39 µg g^−1^ DW, respectively. Sakamoto et al. [56] reported that the α-tocopherol content in *Nostoc commune* Vaucher ex Bornet et Flahault biomass did not exceed 7.7–7.8 µg g^−1^ DW. In this regard, *D. solitarium* MAC–C17 occupies an intermediate position.

The retinol content in cyanobacterial and microalgal biomass varies widely, ranging from 0.01 to 4.28 mg g^−1^ DW [57]. The retinol content in *D. solitarium* MAC–C17 corresponds to the lower range of known values.

Cyanobacteria are known for their ability to accumulate lipids and proteins. From a biotechnological perspective, the most valuable strains are those that combine high lipid and protein content with high biomass productivity. The biomass productivity of Cyanobacteria spans a wide range: 3.7 mg L^−1^ d^−1^ and 30.8 mg L^−1^ d^−1^ for *Leptolyngbya* sp. CENA104 and *Trichormus* sp. CENA77, respectively [58], and 105.58 mg L^−1^ d^−1^ and 127.50 mg L^−1^ d^−1^ for *Oscillatoria* sp. PBGA3 and *Tolypothrix* sp. PBGA1, respectively [59]. The biomass yield of *D. solitarium* MAC–C17 in this study exceeded these values and is comparable to the biomass productivity of species known for their biotechnological value: 115.00 mg L^−1^ d^−1^ for *Chlorella* sp., 140.00 mg L^−1^ d^−1^ for *Ettlia oleoabundans* (S.Chantanachat et H.C.Bold) J.Komárek [59], 210–240 mg L^−1^ d^−1^ for *Nannochloropsis* sp., and 240–280 mg L^−1^ d^−1^ for *Chlorella vulgaris* Beijerinck [60].

The biomass of *D. solitarium* MAC–C17 contained up to 150.05 ± 17.4 mg g^−1^ DW of lipids, with a lipid productivity of 19.26 mg L^−1^ d^−1^. This is significantly higher than for *Leptolyngbya* sp. CENA104 (0.8 mg L^−1^ d^−1^) and *Trichormus* sp. CENA77 (7.3 mg L^−1^ d^−1^) [58], and higher than reported for *Wollea vaginicola* (F.E.Fritsch et Rich) R.N.Singh, which had a lipid productivity of 15.65 mg L^−1^ d^−1^ [17]. It is comparable to *Trichormus variabilis* (Kützing ex Bornet et Flahault) Komárek et Anagnostidis, which had a lipid productivity of 10.48 mg L^−1^ d^−1^ with a higher lipid content (18.55%) in the biomass [61]. *Anabaena sphaerica* MBDU 105 achieved the highest content of 39.21 ± 0.11% DW and lipid productivity of 31.64 ± 0.52 mg L^−1^ d^−1^ under mixotrophic cultivation conditions [42]. The lipid productivity of the well-known oleaginous microalgae *Ettlia oleoabundans* was 34.95 mg L^−1^ d^−1^ [59], while it was 42–63 mg L^−1^ d^−1^ for *Nannochloropsis* sp. and 27–67 mg L^−1^ d^−1^ for *Chlorella vulgaris* [60].

*D. solitarium* MAC–C17 has a high protein content—up to 383.76 mg g^−1^ DW in the exponential growth phase. The protein content in the culture decreased with age. By the 16th day of growth, the protein content decreased twofold compared to the beginning of the experiment in *Anabaena sphaerica* MBDU 105 under photoautotrophic cultivation [42]. Singh [6] described the protein content in *Anabaena* sp. under basal, glucose + nitrate supplemented, and sucrose + nitrate supplemented media on the 16th day of growth as 167.28 mg g^−1^, which is lower than the value for *D. solitarium* MAC–C17. Vijay et al. [46] reported a high protein content similar to *D. solitarium* MAC–C17 in the biomass of *Nostoc* sp. and *Anabaena* sp.—up to 300–350 mg g^−1^ DW. Earlier, El Semary [12] noted that *D. solitarium* is a promising species for enriching fields with nitrogen-containing compounds.

## 4. Materials and Methods

### 4.1. Cyanobacterial Material

The novel strain MAC–C17 was isolated by micropipetting from a freshwater planktonic sample using an inverted Olympus CKX53 microscope (Hachioji, Tokyo, Japan). A sample was collected on 20 September 2024 from the Molochnaya River in the steppe zone of Russia, the Zaporozhye region (N 46°57′05″, E 35°285′20″). A Zeiss Axio Scope A1 microscope (Oberkochen, Germany), equipped with a ×100 oil immersion lens (NA 1.4) and differential interference contrast, was used for light microscopy and photography. Strain was deposited at the Moscow Algoecoengineering Collection MAC (WDCM1344) at K.A. Timiryazev Institute of Plant Physiology RAS.

Observations of the strain lasted from 7 days to 6 months. The strain was cultivated in Z8 medium [62] and in Z8 without nitrogen, since heterocytes can be facultatively absent in several cyanobacterial species in environments with high nitrogen content [35].

### 4.2. DNA Extraction, PCR Amplification and Sequencing

Total DNA from the studied strain MAC–C17 was extracted using Chelex 100 Chelating Resin of molecular-biology grade (Bio-Rad Laboratories, Hercules, CA, USA). The 16S rDNA with a total length of 866 bp was amplified using the pair of primers 8F [63] and B23SR [64]. Amplifications were carried out using PCR mastermixes (ScreenMix, Evrogen, Moscow, Russia). The amplification conditions were as follows: initial denaturation for 5 min at 95 °C, followed by 35 cycles of 1 min denaturation at 94 °C, 45 s annealing at 58 °C, and 1 min 40 s extension at 72 °C, with a final extension for 10 min at 72 °C.

PCR products were visualised by horizontal electrophoresis in 1.0% agarose gel stained with SYBR^TM^ Safe (Life Technologies, Carlsbad, CA, USA). The products were purified with a mixture of FastAP, 10× FastAP Buffer, Exonuclease I (Thermo Fisher Scientific, Waltham, MA, USA) and water. The sequencing was performed using a Genetic Analyser 3500 instrument (Applied Biosystems, Waltham, MA, USA). The internal primers CYA359F [65] and Primer 8 [66] were used for sequencing the 16S rDNA.

Editing and assembly of the consensus sequences were performed by processing the direct and reverse chromatograms in Ridom TraceEdit ver. 1.1.0 and Mega ver. 7 software [67]. The nucleotide sequences of the 16S rRNA gene were aligned using Mafft ver. 7 software and the G-INS-i model [68]. The assembled nucleotide sequence for strain MAC–C17 was included in the alignments along with corresponding sequences of 56 cyanobacterial strains downloaded from GenBank (taxon names and accession numbers are given in Figure 2). The selection of strains for phylogenetic analysis was carried out based on the phylogenetic reconstructions [29,38] and on the inclusion of new sequences from GenBank. The outgroup for the 16S rRNA gene tree comprised two strains of *Raphidiopsis raciborskii* (Wołoszyńska) Aguilera et al. (Aphanizomenonaceae, Nostocales) as a well-supported clade closely related to *Dolichospermum* strains [31,38,69]. The resulting 16S rRNA gene alignment was 869 characters long.

The dataset was analysed using the Bayesian inference method implemented in Beast ver. 1.10.1 software [70] to construct a phylogeny. For the alignment partition, the most appropriate substitution model, shape parameter α, and a proportion of invariable sites (pinvar) were estimated using the Bayesian information criterion (BIC), as implemented in jModelTest ver. 2.1.10 [71]. This BIC-based model selection procedure selected the HKY + I + G model, pinvar = 0.7940, and α = 0.9550. A Yule process tree prior was used as a speciation model. The analysis ran for 7 million generations with chain sampling every 1000 generations. The parameters-estimated convergence, effective sample size (ESS), and burn-in period were checked using Tracer ver. 1.7.1 software [70]. The initial 25% of the trees were removed, and the remaining trees were retained to reconstruct a final phylogeny. The phylogenetic tree and posterior probabilities of branches were obtained from the remaining trees, which had stable estimates of the parameter models for nucleotide substitutions and likelihood. Maximum likelihood analysis was performed using RAxML ver. 7.0.4 software [72]. The GTR + I + G evolutionary model of substitution was used for ML analyses. Nonparametric bootstrap analysis with 1000 replicas was conducted. FigTree ver. 1.4.4 software and Adobe Photoshop CC ver. 19.0 were used for viewing and editing the tree.

### 4.3. Growth Assessment

The cultivation parameters for the strain were based on recommendations from previous studies on Cyanobacteria [73,74,75]. During the preparatory stage, the strain was cultured in Z8 medium [62] until it reached the exponential growth phase. This culture was then used to initiate the experiment.

The assessment of strain growth and biochemical characteristics was conducted in batch (periodic) culture without any intervention in the medium composition throughout the growth period. The experiment was carried out in 250 mL Erlenmeyer flasks containing 150 mL of Z8 medium. The flasks were placed on an orbital ELMI Sky Line Shaker S-3 L (ELMI Ltd., Riga, Latvia) and cultured under constant orbital shaking at 150 rpm.

The cultures grew under standardised conditions at 24 °C, with a light intensity of 70 μmol photons m^−2^ s^−1^, a colour temperature of 4000 K, and a 16:8 h light/dark photoperiod. The light intensity and colour temperature were measured using the Sekonic C-800 spectrometer (Sekonic Corporation, Tokyo, Japan).

During strain growth, biomass increase was monitored based on optical density changes using an IMPLEN Nanophotometer P300 (Implen GmbH, München, Germany) at λ = 720 nm. The specific growth rate (µ) was calculated using Equation (1) [73]:

µ = ln (OD_1_ − OD_0_) (t_1_ − t_0_)^−1^ (1)

where OD_1_ and OD_0_ were the optical density on day 1 (t_1_) and day 0 (t_0_), respectively.

Changes in optical density were used to identify the strain’s growth phases. The DW (g) of the biomass per litter was estimated using a gravimetric method [55].

The biomass productivity (BP, g L^−1^ day^−1^) was estimated using the following equation, where X (g L^−1^) is the concentration of the biomass at the end of the cultivation time t and X_0_ (g L^−1^) is the concentration of the biomass at the beginning of the cultivation:

BP (g L^−1^ day^−1^) = (X_2_ − X_1_) (t_2_ − t_1_)^−1^ (2)

where X_2_ was the dry biomass (g L^−1^) at time t_2_ (day) and X_1_ was the dry biomass (g L^−1^) at time t_1_ (day) [76].

Lipid productivity (LP), protein productivity (PP), retinol productivity (RP), and α-tocopherol productivity (TP) were calculated by the following formula:

LP (PP; RP, TP) (g L^−1^ day^−1^) = BP × L (P; R; T)/100 (3)

where BP—biomass productivity (g L^−1^ day^−1^), L (P; R; T) was the lipid (protein, retinol, α-tocopherol) content of the biomass per DW (%) [17].

### 4.4. Biochemical Parameters Measurement

For strain MAC–C17, all biochemical characteristics were determined during the logarithmic and stationary growth phases. All conditions for sample analysis, as well as instrument settings, have been described in detail in our previous reports: chlorophyll *a*, carotenoids, lipids, protein, vitamin A, vitamin E content, CAT, GPx, SOD, and SDH activity [44,77,78]. Measurement of thiobarbituric-acid-reactive substance content (TBARS), TBA-reactive products during the induction of lipid peroxidation by Fe^2+^ ions (TBARS_in_) and antioxidant activity coefficient (K_AAC_) calculation described in Yakoviichuk et al. [79].

### 4.5. Data Analysis

All measurements were carried out in 3 repetitions. The graphs showed the average values and standard deviation. Statistical analysis was carried out using XLSTAT 2018 software (New York, NY, USA). Statistics were obtained in Microsoft Excel ver. 1903 software (Microsoft Office, Redmond, WA, USA) using Student’s *t*-test. Differences with *p* ≤ 0.05 were considered statistically significant [80]. To generalise and identify patterns in changes in the vitamin content and activity of various enzyme groups in the studied strains, principal component analysis was applied using Statistica ver. 12.0 software. This method simplifies the model system, better describes a complex nonlinear system of relationships, and dynamically evaluates the system’s initial variables [44].

## 5. Conclusions

This study demonstrates that *Dolichospermum solitarium* MAC–C17 exhibits high biomass, lipid, and protein productivity, comparable to the best-known data among Cyanobacteria. This makes it an economically viable feedstock for biodiesel production and for improving soil fertility.

The biomass of *Dolichospermum solitarium* MAC–C17 is rich in chlorophyll *a*, carotenoids, and vitamins, underscoring its value as a raw material for producing specific high-value by-products. The modulation of enzymatic and non-enzymatic antioxidants in *Dolichospermum solitarium* MAC–C17 differed between the exponential and stationary growth phases when cultivated in batch mode. High CAT activity during the exponential phase was replaced in the stationary phase by increased SOD and GPx activities, elevated retinol and α-tocopherol contents, and unchanged carotenoid content. Thus, the strain *Dolichospermum solitarium* MAC–C17 can be a potential producer of highly valuable commercial products. It also holds promise as a prospective subject for the development of antioxidant preparations, solutions aimed at enhancing agricultural productivity, and phytoremediation.

However, the promising results for *Dolichospermum solitarium* MAC–C17 come with caveats and a need for further investigation. The productivity and biochemical characteristics were obtained from specific laboratory settings with controlled temperature, light, and growth media. Successfully scaling up the process will require more research to adapt the technology for real-world production. An additional series of experiments will also be needed to determine the composition of anthropogenic substrates that will be most suitable for phytoremediation using the strain.

## Figures and Tables

**Table 1:** Biochemical and antioxidant characteristics of the strain *Dolichospermum solitarium* MAC–C17 strain in cultures (M ± SD, *n* = 3).

Indicator	Logarithmic Phase, 8th Day	Stationary Phase, 16th Day
Chl *a*, mg g^−1^ DW	6.12 ± 0.34	4.87 ± 0.05
Carotenoids, mg g^−1^ DW	1.33 ± 0.07	1.43 ± 0.04
Chl *a* Car^−1^	1:0.22	1:0.29
Lipids, mg g^−1^ DW	85.15 ± 9.21	150.05 ± 17.4
Protein, mg g^−1^ DW	383.76 ± 24.62	331.22 ± 33.43
Retinol, µg g^−1^ DW	23.15 ± 1.57	31.61 ± 2.14
α-tocopherol, µg g^−1^ DW	31.06 ± 1.48	45.58 ± 1.97
SDH, µmol mg^−1^ min^−1^	1.15 ± 0.11	0.71 ± 0.07
GPx, nmol mg^−1^ min^−1^	97.0 ± 2.36	127.0 ± 9.18
CAT, pmol mg^−1^ min^−1^	3.63 ± 0.12	2.31 ± 0.05
SOD, 10 u.unit mg^−1^ min^−1^	2.72 ± 0.78	3.64 ± 0.54
TBARS, nmol g^−1^ DW	34.52 ± 1.85	27.94 ± 0.83
TBARS_in_, nmol g^−1^ DW	58.44 ± 2.55	51.23 ± 2.74
K_AAC_	0.59	0.55

## Data Availability

The original contributions presented in this study are included in the article. Further inquiries can be directed to the corresponding author.
